# Nodular pyogranulomatous panniculitis due to *Leishmania infantum* infection in a domestic ferret (*Mustela putorius furo*)

**DOI:** 10.1007/s11259-023-10276-x

**Published:** 2023-12-14

**Authors:** Jacobo Giner, Jordi Jiménez, Alex Gómez, Ana Rodríguez-Largo, Carles Juan-Sallés, Cristina Riera, Roser Fisa, Sergio Villanueva-Saz

**Affiliations:** 1 Menescalia Veterinary Clinic, Ismael Merlo Actor, 5, Valencia, 46020 Spain; 2 Els Altres Veterinary Clinic, Carrer del Rosselló, 274, Barcelona, 08037 Spain; 3 Animal Pathology Department, Veterinary Faculty, Universidad de Zaragoza, Miguel Servet 177, Zaragoza, 50013 Spain; 4 Noah’s Path, Arquitecto Santiago Pérez Aracil 30 bajo (centro veterinario), Elche, 03203 Spain; 5 Faculty of Pharmacy and Food Sciences, Universitat de Barcelona, Avda. Joan XXIII s/n, Barcelona, 08028 Spain; 6 Veterinary Faculty, Veterinary Teaching Hospital, Zaragoza University, Miguel Servet 177, Zaragoza, 50013 Spain

**Keywords:** Ferret, *Leishmania infantum*, Panniculitis, Pyogranulomatous, Spain

## Abstract

Leishmaniosis is a vector-borne disease caused by different *Leishmania* species and transmitted by phlebotomine sand flies under natural conditions in Europe. Scientific information related to *Leishmania infantum* in dogs is extensive, where less information is available in cats and other companion animals. Recently, first clinical cases of *L.infantum* infection in domestic ferrrets (*Mustela putorius furo*) have been described. However, clinical information on leishmaniosis in this species is limited A 15-month-old male neutered domestic ferret was presented with chronic weight loss and the presence of coalescent, erythematous and firm subcutaneous nodules in the ventral abdominal subcutis. A fine-needle aspiration of these nodules was performed and the cytological examination revealed a granulomatous inflammation with the presence of macrophages contained a number of oval organisms with an eccentric nucleus and pale cytoplasm, compatible with *Leishmania* spp. amastigotes compatible with Leishmania spp. amastigotes. The nodules were surgically excised and histological examination showed a severe multifocal pyogranulomatous panniculitis. Specific immunohistochemistry and qPCR for *L. infantum* from excised nodules were positive. Additionally, *L. infantum* was cultured and isolated from the nodules by a fine-needle aspiration. An in-house Western Blot test for *L. infantum* was performed in serum sample and a positive result was obtained. This is the first reported case of nodular pyogranulomatous panniculitis due to *L. infantum* infection in a domestic ferret. Further studies are necessary to determine the relevance of domestic ferrets in the transmission of leishmaniosis. The description of new clinical forms of the disease is important as it can assist veterinarians in identifying these new clinical presentations.

## Introduction

Leishmaniosis is an emerging zoonotic disease in European ferrets caused by a protozoan, *Leishmania infantum*, with the description of two cases in Spain in recent years (Giner et al. 2020a, 2021). In Europe, limited epidemiological information is available in *L. infantum* infection in ferrets. An epidemiological study in Spain revealed a 9.0 or 25.5% of *L. infantum* seropositivity in apparently healthy domestic ferrets using enzyme-linked immunosorbent assay (ELISA) or Western Blot (WB) test, respectively (Alcover et al. 2022). This information indicates that the domestic ferrets are exposed to *L. infantum* infection in endemic areas of canine leishmaniosis. Moreover, there are currently no available extended-release topical antiparasitic treatments for sand flies in ferrets commercially, potentially heightening the susceptibility to *Leishmania infantum* infection.

In dogs, the main skin and subcutaneous lesions of leishmaniosis can be diverse and include exfoliative, ulcerative, sterile papular, pustular and nodular dermatitis and sterile nodular panniculitis at the site of parasite inoculation (Papadogiannakis and Koutinas 2015; O´Kell et al. 2010). In general, microscopically lesions are characterized by granulomatous or pyogranulomatous inflammation is detected in canine leishmaniosis. Skin and subcutaneous lesions are usually due to immune complex–mediated (type III) hypersensitivity, although, *L. infantum* amastigotes can induce themselves a pyogranulomatous inflammatory reaction at the site of inoculation (Koutinas & and Koutinas 2014). Occasionally, onychogryphosis can be observed in dogs with leishmaniosis (Koutinas and Koutinas 2014).

In ferrets, there is a limited clinicopathological information related to leishmaniosis (Villanueva-Saz et al. 2021). Only, two cases described an erythematous-papular and ulcerative lesion in an ear pinna and on the lower lip margin, respectively (Giner et al. 2020a, 2021). Hyperglobulinemia caused by polyclonal gammopathy was the most common laboratory abnormality detected in both cases (Giner et al. 2020a, 2021). Both lesions were due to a chronic diffuse pyogranulomatous dermatitis. To increase the knowledge of new clinical forms of leishmaniosis in ferrets, we now document the first case of nodular pyogranulomatous panniculitis due to a *Leishmania infantum* infection in a ferret.

## Materials and methods

### Case history

**Fig. 1:**
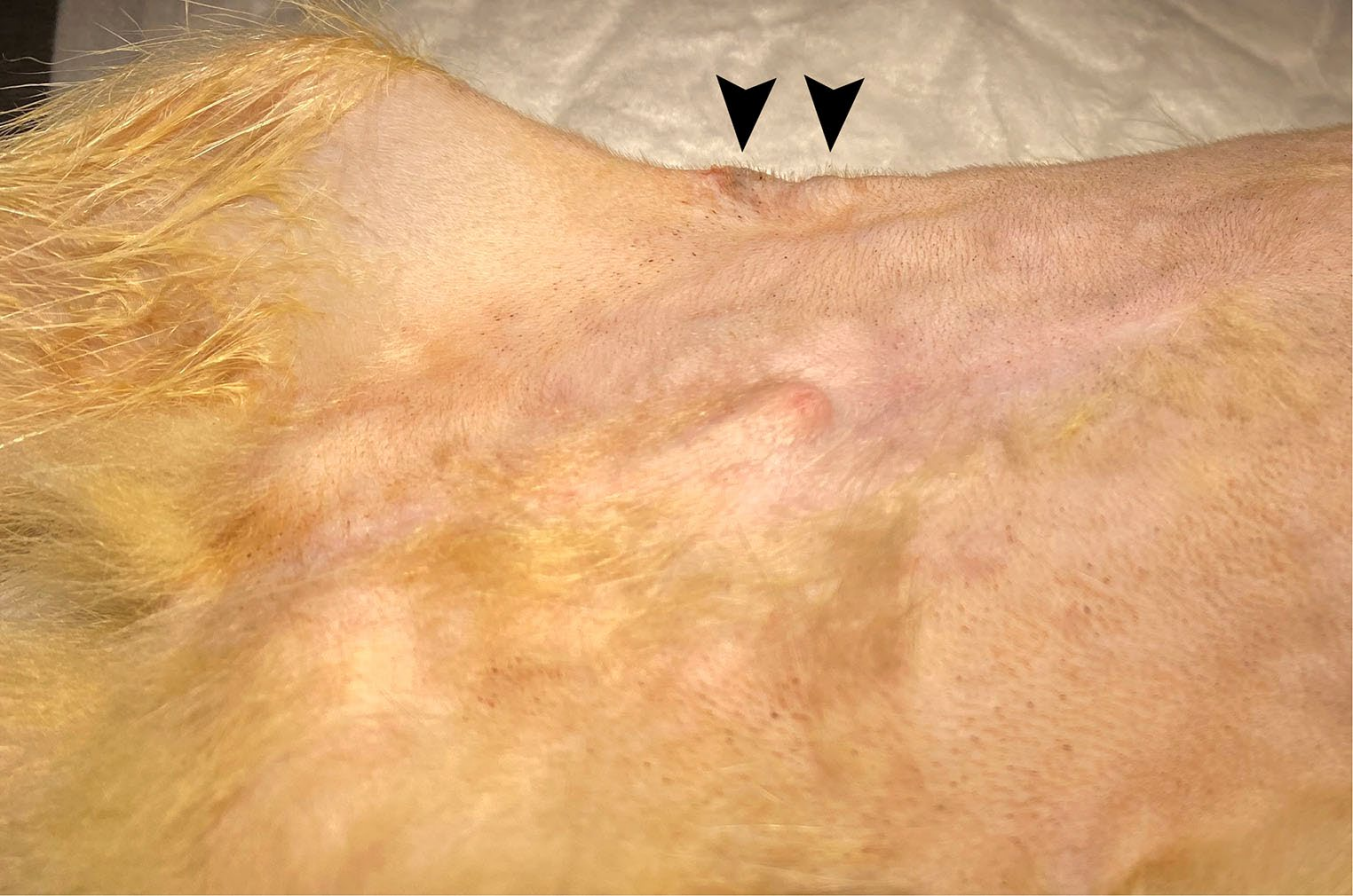
Gross features of nodular pyogranulomatous panniculitis in ventral abdominal subcutis of a domestic ferret. Coalescent, erythematous, and firm subcutaneous nodule (2.5 cm) (arrowhead)

A 15-month-old male spayed domestic ferret from Barcelona (Spain) was clinically evaluated because of the presence of multiple nodules in the ventral abdomen and a progressive weight loss during the previous 10 days. The ferret was adopted at the age of six months old and lived in a house with an indoor lifestyle. It was vaccinated against canine distemper with a single dose of vaccine at seven months of age. On physical examination, it was in good body condition (3/5 body condition), active, alert, normothermic and not dehydrated. Multiple coalescent, erythematous and firm subcutaneous nodules of a variable size in the ventral abdominal subcutis were observed (Fig. 1). Complete blood count, biochemical parameters and serum protein electrophoresis were performed (Table 1. First visit). A fine needle aspirate of a subcutaneous nodule was obtained and stained with Diff-Quick stain for cytological examination. The ferret was premedicated with midazolam 0.2 mg/kg subcutaneously and butorphanol 0.2/kg intramuscularly. Anesthesia was induced with alfaxalone 5 mg/kg intravenously, followed by tracheal intubation (2.0 mm) and maintenance with sevoflurane. Finally, subcutaneous block of the skin with 1 ml 2% lidocaine was administered before skin biopsy.

**Table 1:** Body weight, haematological, biochemical parameters determined in the ferret

	First visit	Second visit (before anti-*Leishmania* treatment, eight weeks later first visit)	Third visit (after anti-*Leishmania* treatment, twelve weeks later after first visit)	Reference range
Body Weight (g)	900	795	865	500–900
Haematology				
WBC (10^9^/L)	11.15	5.75	11.23	2.50–15.80
Neutrophils (10^9^/L)	7.86	2.94	7.18	0.60–10.80
Lymphocytes (10^9^/L)	3.01	1.68	3.05	0.58–10.60
Monocytes (10^9^/L)	0.22	1.06	0.88	0.00-1.12
Eosinophils (10^9^/L)	0.03	0.06	0.10	0.00-0.88
Basophils (10^9^/L)	0.03	0.01	0.02	0.00-0.20
RBC (10^12^/L)	7.63	**5.71**	7.29	6.60-12.18
Haematocrit (%)	**37.4**	**31.4**	37.6	37.50–59.00
Haemoglobin (g/dL)	13.7	**11.2**	**12.2**	12.50–18.20
MCV (fL)	49.0	55.0	51.5	43.60–61.20
MCH (pg)	18.0	19.6	16.7	14.50–20.50
MCHC (g/L)	367	356	325	290–370
RDW (%)	13.8	15.3	16.1	13.10–20.30
Plateles (10^9^/L)	613	725	801	238–910
Blood chemistry				
Glucose (mg/dL)	118	118	129	70.0-144.0
Blood urea nitrogen (mg/dL)	**65**	44	**73**	10.0–45.0
Creatinine (mg/dL)	**2.0**	0.6	0.5	0.2–1.2
Calcium (mg/dL)	**6.8**	**12.8**	9.7	8.0-11.8
Inorganic phosphorus (mg/dL)	**9.3**	6.5	**10.2**	3.6–7.3
Alanine aminotransferase (U/L)	**28**	43	53	48.0-292.0
Alkaline phosphatase (U/L)	34	**5**	20	9.0-120.0
Total bilirrubin (mg/dL)	0.3	0.2	0.2	0.0–1.0
Amylase (U/L)	**16**	**14**	**10**	26.0–36.0
Electrophoretogram of serum proteins				
Total protein (g/dL)	5.4	5.7	6.5	5.2–7.3
Albumin (g/dL)	3.1	4.1	3.4	2.6–4.8
Globulins (g/dL)	2.3	**1.6**	**3.1**	1.8–3.1
Alpha 1 globulins (g/dL)	0.43			
Alpha 2 globulins (g/dL)	0.74			
Beta globulins (g/dL)	0.83			
Gamma globulins (g/dL)	0.30			
Albumin:globulin ratio	1.34			

*Note*: Abnormalities are highlighted in bold

Abbreviations: MCH, mean corpuscular haemoglobin; MCHC, mean corpuscular haemoglobin concentration; MCV, mean corpuscular volume; RBC, red blood count; RDW, red blood cell distribution; WBC, white blood count

### Histopathology and immunohistochemistry labelling to detect the presence of *L. infantum*

A full-thickness subcutaneous nodules biopsy was surgically excised, fixed in 10% neutral-buffered formalin, embedded in paraffin, and 4 μm-thick sections were stained with haematoxylin and eosin (HE). To determine the presence of intralesional *L. infantum* amastigotes, immunohistochemistry was performed using an in-house rabbit polyclonal antibody (Ab) specific for *L. infantum* as described previously (Giner et al. 2020a), with some modifications. Blocking of endogenous peroxidase activity (hydrogen peroxide solution 1%) for 25 min was performed before sections were incubated for 20 min with blocking serum (Vectastain Elite ABC HRP kit (Peroxidase, Universal, cat. no: PK-6200; Vector Laboratories, Inc.)). Thereafter, sections were incubated with rabbit polyclonal anti-*L. infantum* antibody (1 in 500 dilution in TBS) or TBS as a negative control for 3 h at room temperature (RT). Subsequently, sections were incubated with biotinylated secondary Ab (Vectastain Elite ABC HRP Kit (Peroxidase, Universal; cat. no: PK-6200; Vector Laboratories, Inc.)) for 30 min, followed by peroxidase-conjugated avidin-biotin complex for 30 min. Bound antibodies were detected by incubation with diaminobenzidine substrate (Vectastain DAB Substrate Kit, Peroxidase (With Nickel) (cat. no: SK-4100; Vector Laboratories, Inc.)) and sections were then counterstained with haematoxylin. Sections of positive and negative *Leishmania infantum* canine lymph nodes were used as positive and negative controls, respectively.

### Molecular detection of *L. infantum*

Additionally, the presence of *L. infantum* DNA in excised subcutaneous nodules was evaluated using quantitative polymerase chain reaction (qPCR). Nucleic acid was extracted using the MagMAX™ Pathogen RNA/DNA commercial kit (Thermo Fisher Scientific), and the KingFisher Flex System automated magnetic particle processor (Thermo Fisher Scientific), following manufacturer’s instructions. Finally, qPCR was performed on a FAST 7500 cycler (Applied Biosystems) using forward primer (5′-CTT TTC TGG TCC TCC GGG TAG G-3′) and reverse primer (5′- CCA CCC GGC CCT ATT TTA CAC CAA-3′), targeting *L. infantum* kinetoplast minicircle DNA sequences (Alcover et al. 2022). Positive controls (DNA from *L. infantum* MHOM/ FR/78/LEM75 zymodeme MON-1) and negative controls were included in each qPCR analysis.

### Parasite isolation

Aspirated material from a subcutaneous lesion was cultured in Novy-MacNeal-Nicolle (NNN) medium. NNN medium and Schneider medium supplied 100 IU/ mL penicillin and 100 µg/mL streptomycin solution and 10% foetal calf serum was used as described before (Chouihi et al. 2009). Culture wass microscopically assessed every day.

### Serological detection of anti-*Leishmania* antibodies

To evaluate the humoral immune response against *L. infantum*, a blood sample was collected and the detection of specific antibodies anti-*Leishmania* in serum was performed using an in-house WB based on whole antigen of *L. infantum* promastigotes (MHOM/ FR/78/LEM75 zymodeme MON-1), as described previously (Alcover et al. 2022). It was done on 0.1% SDS–13% polyacrylamide gel on a Mini-gel Bio RadSystem. Sera diluted at 1/50 were assayed and a protein A peroxidase conjugate (1/1000 dilution; Pierce) was used. A serum positive was considered when immunoreactivity against the 14 and/or16 kDa *Leishmania* antigen fraction was observed. A positive result is considered when immunoreactivity against *L. infantum* antigen fraction 14 and/or 16 kDa is observed.

## Results

### Case clinical observation, previous therapy and clinicopathological findings before results of confirmatory *Leishmania* techniques

The detection of an increase of blood urea nitrogen, creatinine and inorganic phosphorus was compatible with renal dysfunction. In this sense, no other laboratory abnormalities were detected. Treatment was focused on renal dysfunction control with benazepril at 0.25 mg/kg twice a day PO and fatty acids (omega 3 fatty acids) 80 mg/kg/day for 40 days as anti-inflammatory effect. Eight weeks later, an improvement in previously altered laboratory parameters was detected (Table 1, second visit).

### Results of confirmatory *Leishmania* techniques

**Fig. 2:**
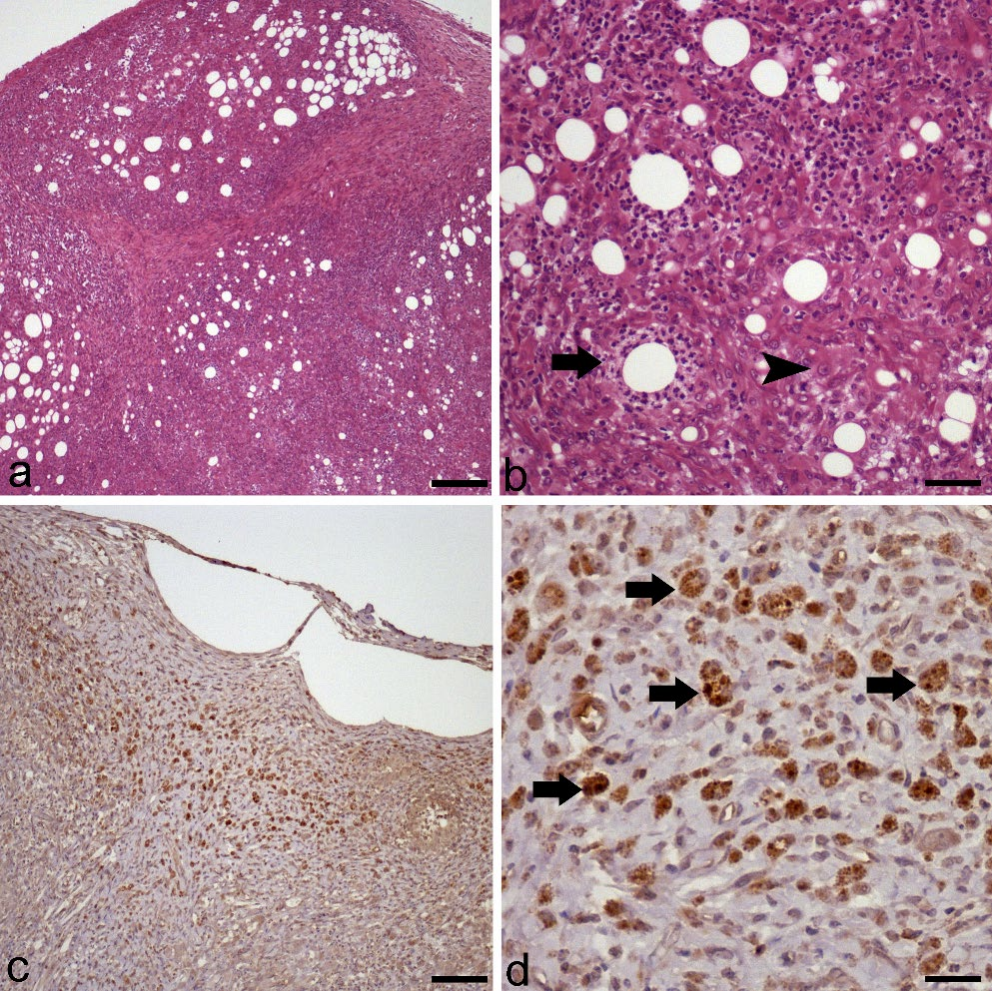
Histopathological and immunohistochemical features of subcutaneous nodule **a**). Diffuse pyogranulomatous panniculitis surrounding and atrophying the adipocytes of the adipose panniculus (HE, bar = 1000 μm); **b**). Foamy macrophages (arrowhead), viables and degenerated neutrophils (arrow) infiltrate and atrophy the adipocytes (HE, bar = 200 μm); **c**). Cells with intracytoplasmic positive granules are distributed throughout the pyogranulomatous lesion (*L. infantum* IHQ, bar = 1000 μm); **d**). Numerous positive *L. infantum* amastigotes within the cytoplasm of macrophages (arrows) (*L. infantum* IHQ, bar = 100 μm)

Cytological examination revealed a low to moderate cellularity comprised of macrophages, fewer multinucleate giant cells and rare degenerate neutrophils in a background of an amorphous eosinophilic material with clear vacuoles. Some of the macrophages contained oval organisms within their cytoplasm. These organisms were characterized by an eccentric nucleus and a small amount of pale cytoplasm, measured approximately 3 to 4 μm in diameter, compatible with *Leishmania spp.* Histological examination revealed the presence of a severe multifocal pyogranulomatous panniculitis (Fig. 2a), where most of the adipocytes in the panniculus were separated, surrounded, or infiltrated by foamy macrophages and viable and degenerated neutrophils (Fig. 2b). High number of oval amastigotes within the cytoplasm of subcutis macrophages stained positively by immunohistochemistry (brown) (Fig. 2c and d). Additionally, NNN medium and Schneider medium was positive after 4 days incubation, achieving parasite isolation. Moreover, qPCR of subcutaneous nodules was positive, in this case, a Ct result of 22 was obtained. This value is in agreement with the result of the in vitro culture, which was positive, allowing the isolation of the parasite, confirming the diagnosis. Anti-*Leishmania* antibodies with a band at 16 kDa were detected in a sampled serum by WB technique.

The diagnosis of pyogranulomatous panniculitis was made based on histopathological results. *L. infantum* was demonstrated as a cause of this pyogranulomatous panniculitis on the basis of cytology, immunohistochemistry, qPCR, in vitro isolation and cultivation, and WB analysis results.

### Case clinical observation and clinicopathological findings after results of confirmatory *Leishmania* techniques

**Fig. 3:**
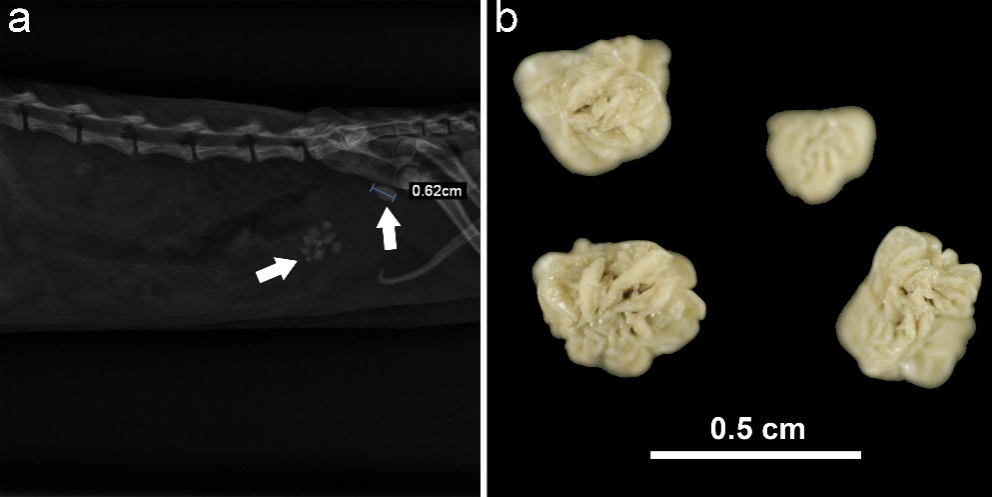
Radiographic and macroscopic findings of uroliths in the bladder and penile urethra **a**). Abdominal radiograph. Right lateral view of the abdomen shows the presence of uroliths. **b**). Calcium oxalate monohydrate urolith

Anti-*Leishmania* therapeutic protocol was established with allopurinol at 10 mg/kg twice a day PO for 6 months. Four weeks later the ferret was examined because of a severe weight loss, apathy, and signs of urinary obstruction represented by repeated attempts to urinate that were unproductive (dysuria), discomfort when straining to urinate and abdominal pain on palpation in the bladder area (Table 1, third visit). Physical examination revealed a little improvement of dermatitis and a worsening of its body condition (2/5 body condition score). Abdominal radiographs revealed the presence of uroliths in the urinary bladder and penile urethra (Fig. 3a).

The patient was ultimately euthanized due to poor prognosis of renal dysnfuction. A post-mortem quantitative urolith analysis indicated that uroliths consisted of 100% calcium oxalate monohydrate (Fig. 3b).

## Discussion

To the best of our knowledge, this is the first case of *Leishmania infantum* associated nodular pyogranulomatous panniculitis in domestic ferrets. In general, seropositive ferrets do not present associated clinic signs or lesions (Villanueva-Saz et al. 2021; Alcover et al. 2022). However, a pyogranulomatous dermatitis has been described recently in two ferrets infected naturally with *L. infantum* (Giner et al. 2020a, 2021). Therefore, this case reinforces the theory that domestic ferrets infected by *L. infantum* can develop pyogranulomatous cutaneous/subcutaneous lesions (Giner et al. 2020a, b) and present new clinical forms in leishmaniosis of ferrets.

Pyogranulomatous dermatitis and panniculitis due to *L. infantum* infection has been well described in dogs (O´Kell et al. 2010) and cats (Matralis et al. 2023). In these species, cases with suspicion of leishmaniosis, cytological and histological examination has been used as an important first step in diagnostic algorithms. In dogs, the inflammatory infiltrate in dermal and subcutaneous lesions caused by *L. infantum* is principally composed by macrophages or macrophages and neutrophils, lymphocytes and plasma cells and less commonly, only neutrophils (Paltrinieri et al. 2016; Torrent et al. 2018). In contrasts, in ferrets, dermal leishmaniosis has been characterized only by a pyogranulomatous inflammatory reaction (Giner et al. 2020a, 2021). In dogs and cats, skin and subcutaneous lesions can be induced by the inflammatory reaction against *L. infantum* amastigotes or deposition of immune complexes (Koutinas and Koutinas 2014; Papadogiannakis and Koutinas 2015). Depending on the pathogenic process, *L. infantum* amastigotes cannot be observed in HE sections from dermal and subcutaneous lesions and immunohistochemistry can be negative. In ferrets, the pyogranulomatous lesions seem to be only due to the inflammatory reaction to the presence of *L. infantum* amastigotes at the inoculation site (Villanueva-Saz et al. 2021), since the HE and immunohistochemistry sections have been positive in all reported cases (Giner et al. 2020a, 2021).

Due to limited information in leishmaniosis in ferrets, in this species, pyogranulomatous dermatitis and panniculitis should be differentiated with dermatophytic pseudomycetomas, *Pseudomonas luteola* infection and cryptococcosis (Halck et al. 2023). To confirm a *L. infantum* associated pyogranulomatous dermatitis or panniculitis in a domestic ferret, the diagnostic protocol should be performed as described in this case. First, a cytology of the mass has to be sampled. If the result is inconclusive, a biopsy should be performed for histopathology and immunohistochemistry, qPCR and/or in vitro isolation study. If the lesions are not accessible, a serological method based on WB, immunofluorescence antibody test or ELISA analysis can be use; however, the presence of apparently healthy seropositive domestic ferrets can difficult the association between the lesion and *L. infantum* infection (Villanueva-Saz et al. 2021). Despite the scarce bibliography, in our experience, histology and immunohistochemistry suggest being the most sensitive diagnostic tests in ferrets.

In ferrets, the presence of xanthinuria has been described with allopurinol administration (Giner et al. 2020b). Additionally, one of the most common adverse effects of allopurinol on the urinary system during treatment of leishmaniosis in dogs is the detection of xanthinuria and xanthine urolithiasis (Torres et al. 2016). In ferrets with clinical leishmaniosis and treated with allopurinol, clinicopathological control of urolithiasis should be performed. Composition of uroliths ought to be considered. In this case, uroliths analysis revealed a chemical composition of 100% calcium oxalate, therefore, urolithiasis was not correlated with allopurinol treatment.

In conclusion, we document the first case of *L. infantum* associated nodular pyogranulomatous panniculitis in ferrets in an endemic area of leishmaniosis. Further studies are necessary to determine the relevance of domestic ferrets in the transmission of leishmaniosis and the different clinical manifestation of infection in this species.

## Data Availability

The data that support the findings of this study are available from the corresponding author upon reasonable request.
